# The prevalence of Coxiella burnetii shedding in dairy goats at the time of parturition in an endemically infected enterprise and associated milk yield losses

**DOI:** 10.1186/s12917-018-1667-x

**Published:** 2018-11-20

**Authors:** José T. Canevari, Simon M. Firestone, Gemma Vincent, Angus Campbell, Tabita Tan, Michael Muleme, Alexander W. N. Cameron, Mark A. Stevenson

**Affiliations:** 1 Asia-Pacific Centre for Animal Health, Faculty of Veterinary and Agricultural Sciences, The University of Melbourne, Corner Park Drive and Flemington Road, Parkville Victoria, 3010 Australia; 2 Instituto de Investigación Animal del Chaco Semiárido, INTA, Leales, Tucumán, 4113 Argentina; 3 Australian Rickettsial Reference Laboratory, Ballerine Street, Geelong Victoria, 3220 Australia; 4 Meredith Dairy, Camerons Road, Meredith Victoria, 3333 Australia

**Keywords:** *Coxiella burnetii*, Coxiellosis, Q fever, Dairy goats, Super shedders, Production losses, Australia

## Abstract

**Background:**

This was a panel study of the prevalence of *C. burnetii* infection in does in an endemic dairy goat enterprise in Victoria, Australia. Our first objective was to determine the prevalence of does shedding *C. burnetii* at the time of parturition and to quantify the concentration of genome equivalents (GE) present in each *C. burnetii* positive sample. Our second objective was to determine the proportion of positive does that were persistent shedders. Our final objective was to quantify the association between *C. burnetii* qPCR status at the time of kidding and daily milk volumes produced during the subsequent lactation.

**Results:**

Vaginal swabs (n= 490) were collected from does at the time of kidding and analysed using a quantitative polymerase chain reaction (qPCR) assay. Shedding of *C. burnetii* was detected in 15% (95% CI: 12% to 18%) of the sampled does. Does were classified as qPCR-negative, qPCR-positive low and qPCR-positive high based on the estimated concentration of GE from the qPCR. Persistent shedding at relatively low concentrations was detected in 20% (95% CI: 10% to35%) of shedding does sampled again at their subsequent parturition. After controlling for possible confounders and adjusting for variation in daily milk yields at the individual doe level, daily milk yields for qPCR-positive high does were reduced by 17% (95% CI: 3% to 32%) compared to qPCR-negative does (*p*= 0.02).

**Conclusions:**

Shedding concentrations of *C. burnetii* were highly skewed, with a relatively small group of does shedding relatively high quantities of *C. burnetii*. Further, high shedding does had reduced milk yields compared to qPCR-negative does. Early detection and culling of high shedding does would result in increased farm profitability and reduce the risk of Q fever transmission.

## Background

Q fever is a zoonotic disease caused by the rickettsia-like bacterium *Coxiella burnetii*. The microorganism occurs in two antigenic variations, phase I and phase II, which differ in the composition of membrane lipopolysaccharides [1]. Q fever affects a wide range of mammalian species among which domestic livestock constitute the main reservoir of infection for humans [2]. Once in the environment *C. burnetii* can survive for prolonged periods of time due to its capacity to differentiate into a spore-like form known as the small cell variant. In the acute phase, clinically infected humans may develop symptoms including high fever, headaches and joint and muscle pain [1]. Life-threatening persistent focalized *C. burnetii* infection(s), mainly endocarditis, can also occur in a relatively small percentage of cases [3]. Goats are frequently identified as a source of Q fever outbreaks in humans [4, 5], as was the case in the outbreak of Q fever that occurred in The Netherlands in 2007 – 2010 where more than 4500 people were infected [6]. Goats are also the livestock species most likely to show clinical signs of disease, which include increased rates of abortion and an increase in the number of stillbirths and weak offspring [7]. An association between subclinical mastitis and *C. burnetii* infection has been reported in dairy cattle [8]. Also, puerperal *C. burnetii* vaginal shedder primiparous cows were found to yield less milk fat compared to negative cows [9]. While histopathological udder lesions have been found in goats artificially infected with *C. burnetii* [10], and an association has been detected between recent *C. burnetii* exposure and milk yield [11], we are aware of no studies that have been published investigating *C. burnetii* shedding at the time of kidding and its association with either subclinical or clinical mastitis or milk yield.

Infected goats can shed large quantities of *C. burnetii* into the environment at the time of parturition. Other documented shedding routes include the milk, feces, urine and saliva, though the amounts of bacteria shed via these routes is believed to be relatively low [12–15]. *C. burnetii* DNA can be detected in vaginal swabs using quantitative polymerase chain reaction (qPCR) techniques, making it possible to identify does that are shedding the infective agent at the time of kidding [16, 17]. Also, qPCR can be used to monitor the presence of *C. burnetii* DNA in bulk tank milk [18–20]. Out of an array of available qPCR assays those targeting the *com1* gene and the insertion sequence *IS1111* are the most frequently used [21]. qPCR assays targeting the *com1* gene have the advantage that the target gene occurs only once within the *C. burnetii* genome, making it possible to quantify the number of genome equivalents in a sample. Compared with the *com1* qPCR the *IS1111* qPCR is more sensitive; an expected feature considering the insertion sequence it targets can occur up to 110 times within the *C. burnetii* genome [22–24]. Antibodies against *C. burnetii* can be assessed using a range of serological techniques of which the immunofluorescence assay (IFA) has the highest diagnostic sensitivity and specificity when used in goats [25].

Australia has one of the highest reported incidence rates of Q fever in humans with between 1.5 and 4.9 notified cases per 100,000 head of population at risk per year [26]. The frequency of Q fever in humans in Victoria has historically been low compared with other states, with incidence rates ranging from 0.3 to 2.0 notified cases per 100,000 head of population at risk per year since 1991 [27, 28]. In 2012–2014 an outbreak of Q fever was associated with a large dairy goat enterprise in Victoria. A total of 24 people were infected; all of them farm staff with the exception of one individual who was a staff family member suspected as having acquired infection via contaminated fomites [29]. Vaccination of all farm staff and elective vaccination of staff contacts with Q-VAX^®;^ (the vaccine used to prevent Q fever in humans in Australia) has, so far, prevented further human cases from occurring. Nevertheless, subsequent investigations carried out on this enterprise showed that coxiellosis was still prevalent among does and that *C. burnetii* DNA as well as specific antibodies to *C. burnetii* were present in bulk tank milk [11].

Long-term studies carried out in dairy cattle, sheep and goat farms where Q fever outbreaks have occurred have shown that infection can persist in herds-flocks for at least two years, with some animals shedding *C. burnetii* in two consecutive parturitions [30–33]. There have been few published reports describing the dynamics of infection in endemically infected herds-flocks. A better understanding of the stages of the production cycle when infection transmission risk is greatest and which age groups or stock classes are responsible for infection transmission provides essential information for the design of herd-flock level Q fever control and eradication strategies.

This was a panel study of the prevalence of *C. burnetii* in an intensively managed dairy goat enterprise related to a human outbreak [29] of Q fever in Victoria, Australia. There were three main objectives. Firstly, to retrieve vaginal swabs from does within 24 hours of kidding and to determine both the prevalence of does shedding *C. burnetii* and the concentration of genome equivalents (GE) present in each *C. burnetii*-positive sample. Our second objective was to select a subset of does identified as *C. burnetii*-positive at the first round of testing and to re-sample them at their subsequent kidding event to determine the proportion that were persistent *C. burnetii* shedders. Our final objective was to quantify the association between *C. burnetii* qPCR status at the time of kidding and both milk yields and somatic cell counts (SCCs) during the subsequent lactation. Results from qPCR testing of vaginal swab samples from aborted does submitted by farm staff were also included in this paper. Throughout this paper we use the nomenclature suggested by Lang (1990) [34] where the term *coxiellosis* is used to describe the presence of *C. burnetii* infection in animals and Q fever describes the clinical condition in humans.

## Results

Does that had vaginal swabs positive for *C. burnetii* were present on all three sampled farms units in all kidding seasons with the exemption of Farm B in the March to April kidding season. Across the four kidding seasons and the three study farms there were 15 (95% CI: 12 to 18) *com1* qPCR-positive does per 100 does at risk. The prevalence of shedding increased from the first to second parities and then decreased for does of parity three and above. Exceptions were the March to April kidding season when shedding was not detected in second parity does and the November to December kidding season when first parity does had a slightly higher prevalence than those in their second parity. When does were grouped by farm and parity the prevalence of shedding was stable over the four kidding seasons ranging from 13% in the September to October season to 17% in the November to December season. For IFA serology the prevalence followed a similar pattern to that of qPCR though the decrease in seroprevalence for second to third parity does was less marked as the decrease in prevalence using the *C. burnetii* qPCR *com1*. When does were grouped by farm and parity the IFA seroprevalence ranged from 24 to 44% in the March to April and June to July kidding seasons, respectively (Table 1).

**Table 1:** Seroprevalence (IFA) and *C. burnetii* shedding prevalence assessed by qPCR in vaginal swabs (*com1*) in recently kidded does on a large dairy goat enterprise in south western Victoria, Australia in 2016

Kidding season	Assay	Does positive / total sampled				Prevalence as no. of positive does per 100 does at risk (95% CI)			
		Farm A	Farm B	Farm C	Total	Farm A	Farm B	Farm C	Total
March to April	*com1*	0/34	11/33	1/22	12/89	0 (0, 10)	33 (20, 50)	4 (1, 22)	13.5 (7.9, 22.1)
June to July	*com1*	16/41	4/55	2/51	22/147	39 (26, 54)	7 (3, 17)	4 (1, 13)	15 (10.1, 21.6)
September to October	*com1*	15/52	2/40	1/42	18/134	29 (18, 42)	5 (1, 16)	2 (0, 12)	13.4 (8.7, 20.2)
November to December	*com1*	17/41	2/40	2/39	21/120	41 (28, 57)	5 (1, 16)	5 (1. 17)	17.5 (11.7, 25.3)
March to April	IFA	13/34	15/33	11/22	39/89	38 (24, 55)	45 (30, 62)	50 (31, 69)	28.3 (21.4, 36.3)
June to July	IFA	9/40	9/55	17/51	35/146	22 (12, 37)	16 (9, 28)	33 (22, 47)	24 (17.8, 31.5)
September to October	IFA	9/51	13/39	9/41	31/131	18 (10, 30)	33 (21, 49)	22 (12, 37)	23.7 (17.2, 31.6)
November to December	IFA	18/41	13/40	9/39	40/120	18 (10, 30)	32 (20, 48)	23 (13, 38)	
33.3 (25.5, 42.2)									
Total	*com1*	48/168	19/168	6/154	73/490	29 (22, 36)	11 (7, 17)	4 (2, 8)	14.9 (12, 18.33)
	IFA	49/166	49/166	46/153	145/486	29 (23, 37)	30 (23, 37)	30 (23, 38)	29.8 (25.9,34)

The distribution of *C. burnetii* GE concentrations in vaginal swabs was highly skewed. While the majority of does that were *C. burnetii* qPCR *com1* positive shed relatively low quantities of bacteria, the concentration of a *C. burnetii* GE in a small number of does (*n* = 14) was above > 10^3^ and as high as 10^9^ GE per µL of sample. There was a fair agreement between serology and shedding status after adjusting for bias (PABAK 0.37; 95% CI: 0.28 to 0.45). Nevertheless, 39 out of 71 does that were *C. burnetii* qPCR *com1* positive returned a negative result when tested with the IFA. The risk of a doe being IFA positive if she was shedding more than 10^3^ GE per µL as measured by the qPCR *com1* assay was 2.6 (95% CI 1.8 to 3.8) times the risk of a doe that was negative to the qPCR *com1* assay (Table 2). Log10 transformed *C. burnetii* GE concentrations for does of parities 1, 2, 3 and 4+ identified as positive were not statistically significantly different (Kruskall-Wallis test statistic 5.45; *p* = 0.14).

**Table 2:** Levels of shedding (GE/ *μ*L)determined by qPCR *com1* assay and serological status (IFA) in an endemically infected dairy goat enterprise in Victoria, Australia

Shedding level	Term kidders				Aborted goats^a^
(GE per µL)	IFA +	IFA -	IFA missing	Total	Total
Negative	113	302	2	417	12
1-10^3^	22	35	2	59	4
>10^3^	10	4	-	14	10
Total	145	341	4	490	26

^a^No serology was available for aborted goats

GE = genome equivalents

Of the 40 does that were *C. burnetii* qPCR *com1* positive in 2016 and re-sampled when they kidded again in 2017, none were positive when tested with the *com1* assay (0%; 95% CI 0% to 9%) whereas 8 (20%; 95% CI: 10 to 35%) were positive when tested with the *IS1111* assay. Out of the 26 vaginal swabs from aborted does submitted for *com1* qPCR testing, 14 (54%; 95% CI: 35% to 71%) returned a positive result.

Mean daily milk volume yield for all does during the follow-up period (i.e. lactations starting with the kidding event at which does were sampled and up to 305 days in milk) was 3.03 (95% CI: 3.02 to 3.04) litres. After adjusting for the effect of farm, season, parity and days in milk and using a random effect term at the individual doe level, does in the qPCR-positive high group produced 0.53 (95% CI 0.08 to 0.98) litres less milk per day than does in the qPCR-negative group (Table 3). Daily milk yields for does in the qPCR-positive low group were similar to daily milk yields in the qPCR-negative group. No statistically significant association at the *p*≤ 0.05 level was found for qPCR status and SCC.

**Table 3:** Australia in 2016. Multiple linear regression model outputs of factors influencing daily milk yields in does on a large dairy goat enterprise in south western Victoria

Variable	Coefficient (SE)	*t*	*p* value	95% CI
Intercept	2.38 (0.11)	21.26	< 0.001	2.15 to 2.59
qPCR status:				
Negative	Reference		-	-
Positive low	-0.08 (0.12)	-0.68	0.495	-0.33 to 0.16
Positive high	-0.53 (0.23)	-2.33	0.02	-0.98 to -0.08 ^*a*^
Parity:				
1	Reference		-	-
2	0.34 (0.10)	7.29	< 0.001	0.54 to 0.94
3	1.22 (0.11)	10.7	< 0.001	0.99 to 1.44
4+	0.71 (0.13)	5.5	< 0.001	0.46 to 0.96
Kidding season:				
March to April	Reference		-	-
June to July	0.06 (0.11)	0.49	0.62	-0.17 to 0.28
September to October	0.53 (0.12)	4.52	< 0.001	0.3 to 0.77
November to December	0.54 (0.12)	4.57	< 0.001	0.3 to 0.77
Farm:				
A	Reference		-	-
B	0.24 (0.10)	2.34	0.02	0.04 to 0.44
C	-0.71 (0.11)	-6.72	< 0.001	-0.92 to -0.5
Random effect	SD			
Goat id	0.82			
Within goat temporal correlation with first order autoregressive structure (AR1): 0.23				

AIC: 343081.1

^a^Interpretation: After adjusting for the effect of parity, kidding season, days in milk and farm, daily milk yields from does that were qPCR-positive high were 530 (95% CI 80 to 980) mL less than does that were qPCR negative. The random effect at the goat level accounts for repeated measures and individual animal variability.

SE: standard error.

CI: confidence interval

## Discussion

The results of the present study show that under intensive management conditions coxiellosis in dairy goats can become endemic, with a relatively high prevalence of *C. burnetii* shedding does at parturition after several years from introduction of disease in the herd. Vaccination with a phase I vaccine is regarded as an effective control measure for *C. burnetii* infection in dairy goats [35]. However, no Q fever vaccines are licensed for use in animals in Australia. Efforts are currently underway to develop an autogenous vaccine to immunize animals in the enterprise where the present study was carried out. A study by Astobiza et al. (2010) [36] showed treatment of pregnant sheep with oxytetracycline did not reduce the risk of *C. burnetii* shedding at lambing. The impact of changes in management practices (like the use of prolonged lactations to reduce the number of kidding events per year) on disease transmission have been modeled by Bontje et al. [37]. Predictions from this model suggest that while these interventions can lead to a reduction in the amount of environmental contamination, they alone are not sufficient to allow the disease to be eradicated. While the study of Bontje et al. [37] provides useful insights into the effectiveness of coxiellosis control methods in a European herd management context, its ability to be applied to Australian dairy goat management conditions is limited mainly due to the complex seasonal kidding patterns that are feature of intensively managed dairy goat herds in Australia. There is a clear need for herd-flock managers to have access to tools and/or management strategies to allow coxiellosis to be controlled and/or eradicated in livestock farms in Australia. Ideally, the relative efficacy of recommended tools should be based on empirical evidence. The results presented in this paper partially address this need.

Our results show that while most qPCR-positive does shed relatively low concentrations of *C. burnetii* in their vaginal fluids at the time of parturition, a small proportion shed very high concentrations. Interestingly, the small proportion of does that were shedding high concentrations of *C. burnetii* (which we term ‘super shedders’) shed similar concentrations to does that had aborted. The phenomenon of super shedders has previously been described for coxiellosis as well as for other infectious diseases of livestock. In Germany, a single *C. burnetii* super-shedding ewe that lambed in a farmers’ market was the source of a Q fever outbreak that resulted in 299 infected people [38]. Capparelli et al. [39], studied *Brucella abortus* shedding patterns in the milk of water buffaloes and found that while the majority of *Brucella abortus*-positive buffalo were shedding low concentrations of bacterium a small proportion shed very high concentrations. Similar findings were reported by Omisakin et al. [40] in a study of *Escherichia coli* O157 in cattle in the United Kingdom. In their cross-sectional study Omisakin et al. estimated that approximately 97% of *Escherichia coli* O157 recovered from the environment was attributable to 9% of animals that were shedding.

In a cross-sectional study following an outbreak of coxiellosis in goats in France de Cremoux et al. [41] quantified the number of *C. burnetii* GE recovered in vaginal swabs in recently kidded does using the *IS1111* assay. de Cremoux et al. [41] showed that between 6% and 33% of does across three flocks were high shedders (greater than 10^6^ bacteria per swab). While the findings of de Cremoux et al. are not directly comparable with ours because GEs were estimated using a different assay and results were reported as absolute GE counts (as opposed to concentrations), it is evident that in both studies the distribution of GE estimates is skewed, meaning that while most qPCR-positive does shed small amounts of *C. burnetii*, a substantially smaller fraction shed large amounts. If does contributing to the majority of environmental contamination can be identified and culled before either aborting or kidding this could have a major impact on reducing the level of environmental contamination and the probability of disease transmission.

While super shedders are likely to play an important role in the transmission of disease, persistent shedders can perpetuate coxiellosis transmission. Of the 40 does that tested positive to *com1* in 2016 and were tested again in 2017, all were negative to the *com1* assay and 20% (*n* = 8) were positive to the *IS1111* assay. Due to the superior sensitivity of the *IS1111* assay compared with the *com1* assay our inference is that the level of shedding in the persistently shedding group of does was low. Berri et al. (2005) [42] conducted a longitudinal study in a goat herd in France that had experienced an abortion outbreak due to coxiellosis and found no PCR positives in vaginal swabs when does were sampled at the subsequent kidding season. This finding led the authors to hypothesize that *C. burnetii* shedding by infected does was limited to one parturition. We note however, that vaginal swabs in that study were obtained two weeks after parturition, which could have markedly reduced the likelihood of detecting *C. burnetii* shedding does. Later, in an investigation of an outbreak of abortions in another dairy goat herd in France, Berri et al. (2007) [31] found that 26% of the does that were *C. burnetii* shedders at the time of kidding had also been shedders at their previous kidding. *C. burnetii* GE concentrations for the repeat-shedding does were not reported. In our study we show that infected does can shed *C. burnetii* over at least two consecutive parturitions. A weakness of previously published research in this area has been that the change in *C. burnetii* infection status of individual does from one parturition to the next has not been unambiguously reported. Instead, authors have simply documented the group-level prevalence of *C. burnetii* for groups of consecutively kidding does. This approach is likely to misrepresent the true proportion of does that are persistent shedders due to (among other factors) individual herd replacement and culling rates. We conclude that if one was attempting to eradicate Q fever from a dairy goat herd, priority should be placed on identifying and isolating high shedders as opposed to identifying and removing persistently shedding does. Our reasoning for this is two-fold. Firstly, the proportion of persistently shedding does is relatively small and secondly the quantities of *C. burnetii* shed by persistently shedding does is relatively small. Our results show that for monitoring purposes the *IS1111* assay is preferred over the *com1* assay due to its superior sensitivity for detecting relatively low levels of *C. burnetii* DNA.

Does presented for sampling were those that had kidded during the previous 24 hours. Due to logistic limitations we were unable to determine the exact time of kidding for each doe. For this reason it is possible that detected differences in *C. burnetii* shedding quantities were influenced by the interval from kidding to the time of sampling, with higher concentrations of *C. burnetii* likely to be shed closer to the time of delivery, leading to missclassification of individual doe qPCR category assignment. An argument against this explanation is the negative association between daily milk yields for *C. burnetii* qPCR-positive high does as discussed below. Abortion rates reported by the herd managers in this enterprise ranged from 2 to 8% across the three farms and four kidding seasons. It cannot be ruled out that abortion causes other than coxiellosis also played a role in fetal loss in these herds. Throughout 2016-2017 14 vaginal swab samples from the 26 does that aborted returned a qPCR *com1* positive result. The concentration of *C. burnetii* GE found in positive samples from aborted does was high (Table 2) and similar to those found in a prospective study of sheep and goat abortion cases in Ontario, Canada [43]. An area of interest for future work on this farm would be to investigate abortion causes and the extent to which coxiellosis alone, or in combination with, other abortion-causing agents is responsible for reproductive losses.

The agreement between serology and qPCR was not substantial, which is consistent with findings from other studies [41, 44–46]. However, the agreement improved in does that were shedding high concentrations of *C. burnetii* (Table 2). Out of the 14 goats shedding above 10^3^ GE/ *μ*L, 10 had a positive result to IFA. The reasonably strong agreement between qPCR and serology for high shedder does indicates that there may be some use for serology as a means for detecting high shedder does in endemically infected herds. We note that in the present study vaginal swabs and blood samples were collected at the same time (within 24 hours after kidding) hence it cannot be assumed that high shedders could actually be detected using serology before kidding. A useful area of future research would be to collect blood samples for IFA serology from does entering their last third of gestation and to compare the IFA results to qPCR *com1* results from vaginal swabs taken at the time of kidding. Detection of *C. burnetii* in whole blood samples by qPCR is a possible alternative to serology for this purpose, which could have the additional benefit of being a method for detecting super shedders in vaccinated herds.

With regards to the impact of *C. burnetii* infection on milk yield we identified that daily milk volume yields were lower in does that comprised the qPCR-positive high group compared with qPCR-negative does. Given average daily milk yields in these herds were in the order of 3 litres, our inference is that *C. burnetii* infection in the relatively small proportion of does classed as high shedders results in a substantial (i.e. 3 to 32%) reduction in daily milk yields. In a study that assessed seroconversion dynamics at the time of kidding Muleme et al. [11] found that does that had seroconverted to phase I IgG produced 0.2 L of milk per day more than does with no protective antibody response in the first 9 weeks of lactation. We hypothesize these results represent two sides of the same coin, the drop in milk yields associated with *C. burnetii* infection found in the present study relates to the gain in daily milk yields observed by Muleme et al. in does with a protective immune response. Infection with *C. burnetii* in goats can lead to placentitis, endometritis and mild lesions in the mammary gland [10], and these conditions provide plausible biological explanations for the milk losses identified in this study. In cows, Barlow et al. [8] found an association between the presence of *C. burnetii* in individual milk samples and increased SCCs. In the present study, no statistically significant association between individual SCCs and *C. burnetii* qPCR results in vaginal swabs was found. We note, however, that only one individual SCC estimate was available per doe per lactation. Testing for presence of *C. burnetii* in individual milk samples, as opposed to vaginal swabs, and controlling for presence of most common microorganisms that cause mastitis in goats would be a more appropriate way of addressing the question on whether *C. burnetii* can cause subclinical mastitis in goats. This is the first time a negative association between *C. burnetii* infection status and milk yield in dairy goats has been reported.

## Conclusions

Shedding concentrations of *C. burnetii* were highly skewed, with a relatively small group of does shedding relatively high quantities of *C. burnetii*. Further, high shedding does had reduced milk yields compared to qPCR-negative does. Agreement between serology and shedding status was improved in does shedding high concentrations of *C. burnetii*. Future studies should aim at developing improved methods for early detection of high shedding does. Eradication of *C. burnetii* from intensively managed dairy goat herds should have a positive impact on herd profitability (in terms of increasing daily milk yields) as well as reducing the likelihood of dairy goats being a source of *C. burnetii* infection for humans.

## Methods

### Study design and study population

The source population for this study were does on three of the four farm units that comprise a large dairy goat enterprise in south western Victoria, Australia. The eligible population were does that kidded during the period 1 March to 31 December 2016. At the start of the study there were up to 1500 does milking in each of the three study herds. The majority of does in each herd were Saanen with smaller numbers of Toggenburgs and Alpines. Does were housed in sheds using a deep litter system and fed a mixture of concentrates and roughages *ad libitum* throughout the lactation. In each of the herds mating was timed so that does kidded in four batches throughout the year (March to April, June to July, September to October and November to December). Each herd was visited once weekly during each of the four kidding seasons and vaginal swab and whole blood samples were taken from each doe that had kidded during the previous 24 h, leading to a total of 490 samples from full-term kidding does. A sub-sample of does (*n* = 40) identifed as *C. burnetii*-positive from the 2016 sampling had vaginal swabs taken again at their subsequent kidding event in 2017. Throughout 2016 and 2017, an additional 26 vaginal swabs from aborted does were obtained by farm staff and submitted for qPCR *com1* testing. The volume of milk produced by each doe for each day of lactation was measured and recorded using the milking machine used on each farm. Individual doe milk samples were collected on four occasions throughout the year and submitted for individual doe somatic cell count estimation. These details were collated with each doe’s biographical data, with most of the does included in the study having at least one SCC estimate. Biographical data for each doe, the dates of sample collection and the results of vaginal swab and blood tests were stored in a relational database.

DNA from each of the vaginal swab samples was extracted using a HiYield Genomic DNA Mini Kit (Real Biotech Corporation) according to the manufacturer’s instructions. Real time PCR analyses targeting the *com1* gene were carried out using the methodology described by Lockhart et al. (2011) [47]. The number of *C. burnetii* GE per µL of sample was estimated using a standard curve described by Muleme et al. (2017) [48]. Vaginal swabs from the 40 does identified as positive to the *com1* qPCR in 2016 and resampled in 2017 were tested using both the *com1* and *IS1111* qPCR. The *IS1111* qPCR assay was carried out following the methodology described in [49]. Only samples presenting the typical amplification curve and with a Ct (cycle threshold) value below 40 were considered positive. High-pure water served as the negative control for the qPCR assays. The *com1* amplicon cloned into a plasmid and DNA extracted from Vero cell cultures of *C. burnetii* Nine Mile RSA439 (Phase II, Clone 4) were used as positive controls for the *com1* and *IS1111* assays, respectively. Serological testing was carried out using the methods described by [25] using a 1/160 dilution cut-off. For IFA negative controls serum from New Zealand goats (a country declared free of *C. burnetii* by the World Organisation for Animal Health) was used. Serum samples from two goats from a known *C. burnetii*-positive farm that tested positive to the complement fixation test (CFT) were used as IFA positive controls.

### Statistical analyses

#### Impact of qPCR status on daily milk yields

Individual daily milk volume records of up to 305 days in milk were available for 462 of the 490 does sampled in 2016 (*n* = 110,024 records in total). Daily milk volumes greater than 15 litres (*n* = 311) were deemed implausible and were discarded from the analyses. Does were grouped into three categories based on the estimated concentrations of *C. burnetii* present in their vaginal swab samples. The number of *C. burnetii* GE per µL present in each qPCR-positive vaginal swab sample was plotted as a frequency histogram. Does shedding *C. burnetii* concentrations greater than the third quartile plus 1.5 times the interquartile range (i.e. outliers) comprised the first group (*n* = 14). Does shedding less than this threshold comprised the second group (*n* = 56), and those that were *C. burnetii*-negative comprised the third group (*n* = 392). In the remainder of this paper we use the terms qPCR-positive high, qPCR-positive low, and qPCR-negative (respectively) to refer to does in each of these categories. The mean Ct values for groups qPCR-positive low and qPCR-positive high were 34.9 (95% CI: 34.1 to 35.6) and 23.9 (95% CI: 19.7 to 28.2), respectively. To compare daily milk yields in the first 305 days of lactation among the three *C. burnetii* qPCR *com1* status groups a generalized additive mixed-effects model was developed. Parity, kidding season, farm and days in milk were included as fixed effect terms in the model to adjust for their potentially confounding effect on daily milk yield. A penalized spline term was used to account for the non-linear association between days in milk and daily milk yield. Lack of independence in the data arising from repeated measurements of milk yield for each doe were accounted-for by including a random effect term at the individual doe level. Several options for covariance structure of the random effect term were tested and compared using the Akaike Information Criterion (AIC), and the best fitting of these, a first order autoregressive (AR1) covariance structure was used to account for autocorrelation of error terms [50]. The final model was of the form:

$$\begin{aligned} {MY}_{ij} &=\! \beta_{0} + \beta_{1} {Status}_{i} + \beta_{2} {Parity}_{i} \,+\, \beta_{3} {Season}_{i} \,+\, \beta_{4} {Farm}_{i}\\ & \quad+ \beta_{5} s({dim}_{i}j) + D_{i} +\epsilon_{ij} \end{aligned} $$

$$D_{i} \sim N\left(0,\sigma^{2}_{doe}\right), \hspace{1cm} \epsilon_{ij} \sim N\left(0,\sigma^{2}\right) $$

The outcome variable *MY*_*ij*_ is the milk yield (in litres) of the *i*^*th*^ doe on the *j*^*th*^ day of lactation. Daily volume of milk produced was a function of an intercept term *β*_0_ and regression coefficients *β*_1_...*β*_5_ representing the effect of the 3-level categorical variable *C. burnetii* qPCR *com1* status, (described above), parity of each doe at the time of kidding (first, second, third, and fourth parity or greater), kidding season in which the doe kidded (March to April, June to July, September to October and November to December), farm (’A’, ’B’ and ’C’) representing which of the three farms a doe was located for the duration of her lactation and days in milk (an integer ranging from 1 to up to 305), respectively. The normally-distributed random effect for each doe is represented by the term *D*_*i*_ and *ε*_*ij*_ is the residual error (assumed to be independent and following a Normal distribution).

#### Impact of qPCR status on somatic cell counts

Individual SCC records were available for 401 of the goats sampled (one test per goat). Following the same methodology described in “Impact of qPCR status on daily milk yields” section, does were grouped as qPCR-positive high (*n* = 11), qPCR-positive low (*n* = 53), and qPCR-negative (*n* = 337). To compare SCCs among the three *C. burnetii* qPCR *com1* status groups a multiple linear regression model was developed. SCCs were transformed to a log (natural) scale to meet normality assumptions. The variables parity, kidding season, milk yield on the day of the test (available in the database aforementioned), days in milk and farm were included as fixed effect terms to account for their potentially confounding effect on SCCs. The final model was of the form:

$$\begin{aligned} {}LogSCC{i} &= \beta_{0} + \beta_{1} {Status}_{i} + \beta_{2} {Parity}_{i} + \beta_{3} {Season}_{i}\\&\quad + \beta_{4} {Yield}_{i} + \beta_{5} {Farm}_{i} + \beta_{5} {dim}_{i} + \epsilon_{i} \end{aligned} $$

$$\epsilon_{i} \sim N\left(0,\sigma^{2}\right)$$

The outcome variable *LogSCC*_*i*_ is the natural log SCC for the *i*^*th*^ doe. The log transformed SCCs were a function of an intercept term *β*_0_ and regression coefficients *β*_1_...*β*_5_ representing the effect of the 3-level categorical variable *C. burnetii* qPCR *com1* status, (described above), parity of each doe at the time of kidding (first, second, third, and fourth parity or greater), the season in which the doe kidded (March to April, June to July, September to October and November to December), farm (’A’, ’B’ and ’C’), milk volume produced on the day of the SCC test and days in milk at the time of SCC test. Finally, *ε*_*ij*_ represents the residual error term (assumed to be independent and to follow a Normal distribution).

For both regression models, all explanatory variables were forced into the model irrespective of their statistical significance on the basis that they were considered a priori to be potential confounders of the association of interest. Frequency histograms of the residuals and plots of the residuals versus predicted values were developed to check that the assumptions of normality and homogeneity of variance had been met. The observed data was superimposed over a line plot of model predictions to visually assess model fit. Significance level was set at *α* = 0.05. Statistical analyses were carried out using R version 3.4.3 [51] and packages ’gamm4’ [52], ’lme4’ [53] and ’epiR’ [54].

## Abbreviations

AIC: Akaike information criterion
AR1: First order autoregressive (covariance)
CI: Confidence interval
CFT: Complement fixation test
Ct: Cycle threshold
DNA: Deoxyribonucleic acid
GE: Genome equivalents
IFA: Immunofluorescence assay
IgG: Immunoglobulin G
PABAK: Prevalence-adjusted and bias-adjusted kappa
qPCR: Quantitative polymerase chain reaction
SCC: Somatic cell count
SE: standard error
